# Supplementary material for: Piezo-generated charge mapping revealed through direct piezoelectric force microscopy
Source: Nat Commun. 2017 Oct 24;8:1113. doi: 10.1038/s41467-017-01361-2 (PMC5653648; doi:10.1038/s41467-017-01361-2)
Supplement: Supplementary file 1 — Supplementary Information [file 41467_2017_1361_MOESM1_ESM.pdf]

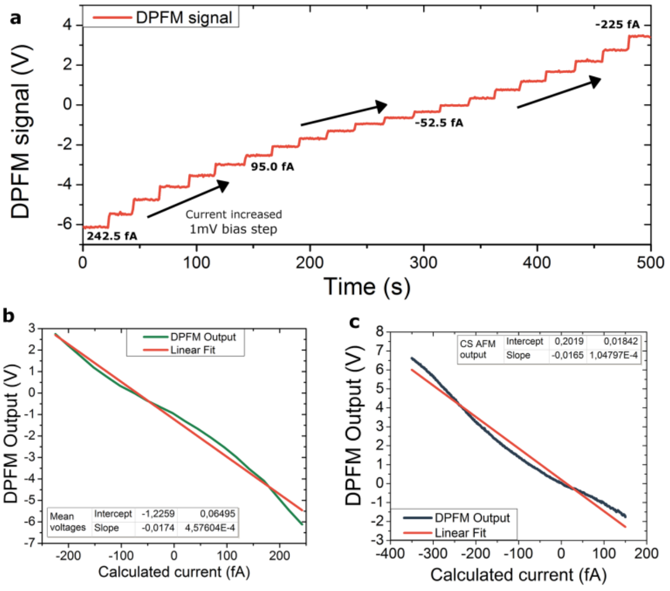

Supplementary Figure 1: Calibration of operational amplifiers. a, DPFM Output Signal (V) vs Time (s) where each applied bias was increased in 1mV steps. From the voltage applied and knowing the test resistor value used for calibration, we can find the exact calculated current that passes through the resistor. The resulting calculation is plotted as a DPFM Output (V) vs Calculated current (fA) in **b**. A linear fit is used to find the slope of the curve, which is the first of both experimental obtained gains. **c** corresponds to the DPFM Output (V) vs Calculated current (fA), data obtained by changing the applied bias through a 600 seconds, 600 points, curve. A linear fitting provides a second value for the calibration process. The amplifier was populated with a feedback resistor of 1TeraOhm, with part number MOX112523100AK from Ohmite, which is commercially available and has an accuracy of 1%. Two subsequent ultra-low-noise voltage amplifiers from Analog Devices Inc where selected, with part number AD8429BRZ. Each of the amplifiers is populated with a metal film resistor of 780 Ohm having with uncertainty of 1%. Even though all the components used are state of the art, there is a slight difference between the theoretical gain calculation of 72,25 x 10^{12} V A^{-1} and the real gain of the amplifier. In order to quantify this deviation, an experimental gain calculation was obtained. We selected a 40GOhm resistor, part number MOX-1125-23-4008J to perform a calibration step process. The resistor was connected to the DC voltage source of the AFM while at the same time, a multimeter, with part number HP 3457A was used to exactly measure the voltage applied. Through Ohms law, a theoretical current can be calculated, by simply dividing the applied bias by the 40 Gohm resistor. The calculated current is used to calibrate the output voltage signal of the amplifier. Using a linear fit, we found the first value for the amplifier calibration constant, as being -0,0174 V fA^{-1} (-17,4 10^{12} V fA^{-1}) with standard error of 0,000457 V fA^{-1}. The linear fit from **c** give us the second calibration constant, which is -0,0165 V fA^{-1} with standard error V fA^{-1}. The final value for the amplifier calibration constant is found by calculating the average of each mean value, which gives us a value of -0,0169 V fA^{-1} with an standard error of 0,000172 V fA^{-1}. To such error, we must add a 5% additional error that comes from the uncertainty in the test resistor used. The final error is found to be 0,00101 V fA^{-1}.

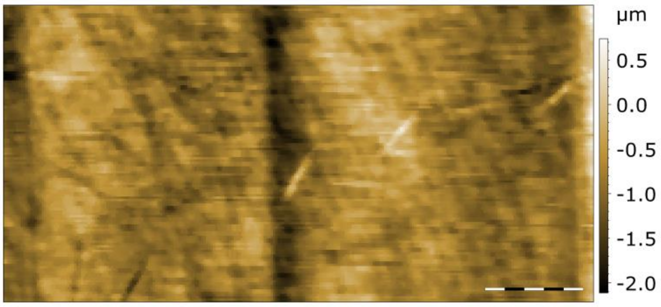

Supplementary Figure 2: Topography information of periodically poled lithium niobate. Topography image obtained simultaneously with the frames showed on Figure 2, scale bar 7,5 µm. Even with the high applied loading force the feedback of the AFM is capable of resolving the sample topography. Along with the four frames presented in Figure 2 which are: DPFM-Si, DPFM-So, PFM amplitude and PFM phase; we can also obtain standard topography images.

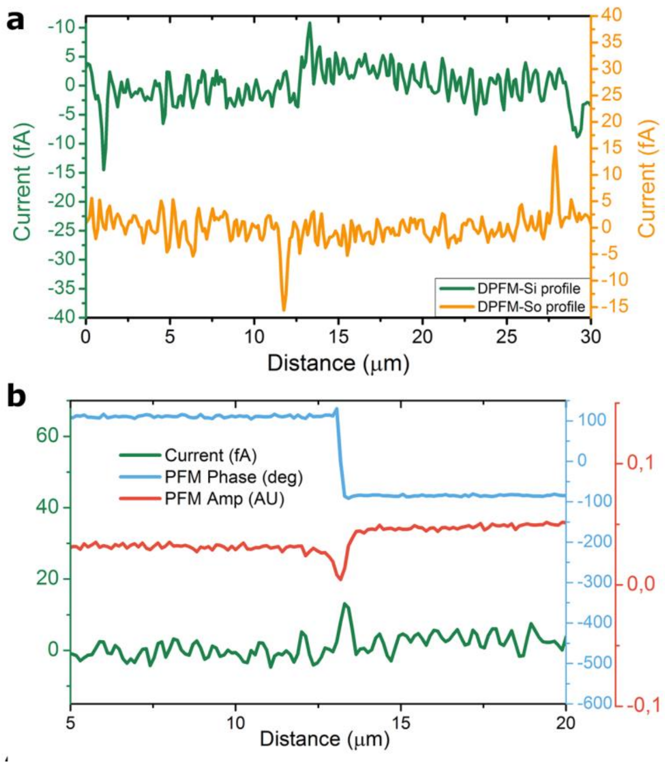

Supplementary Figure 3: Extracted profiles. a DPFM-Si (green) and DPFM-So (yellow) profiles vs Distance (μm), obtained extracting a single profile from the images plotted in Figure 2a and 2b. It is seen that the peak values recorded are ±15fA while the background signal variations are below 1,5fA. **b**, DPFM-Si (green), PFM phase (blue) and PFM amplitude (red) profiles vs Distance (μm), obtained from images Figure 2a and 2c. It is found that a full electromechanical and piezoelectricity generation mechanism is under covered with the proposed mode. From the profiles we can see that the current is mostly collected in the domain walls regions, while in the single domain regions there is a little background signal, a background that is near one order of magnitude less compared to top peak values. From the profiles, top peaks represent that a positive charge is generated, while bottom peaks represent that a negative charge is generated. The peaks delimitate domains position, which have a value of ±15fA. The variations obtained in the single domain areas-areas between peaks- can be caused by multiple phenomena, from Johnson noise, to surface screening charges, sample tilting, topography artifacts..etc. Investigation on the origin of these background signals should be the object of further research. However it is important to note its values compared to the recorded current signal at domain walls, which have been calculated as standard deviation values of 1,0 and 1,3 fA for DPFM-So and DPFM-Si, respectively. The peaks from DPFM-Si and DPFM-So do not coincide because of the hysteresis of the AFM piezo scanner tube, which changes upon the applied load. There is a slightly shift to the right in the DPFM-Si signal, compared to the PFM phase and PFM amplitude signal. This effect can be attributed to the specific low bandwidth of the amplifier, 1-3 Hz, which causes a delay in its output signal. With such mode, a full understanding of the electromechanical and piezoelectricity behavior of a given ferroelectric specimen can be obtained.

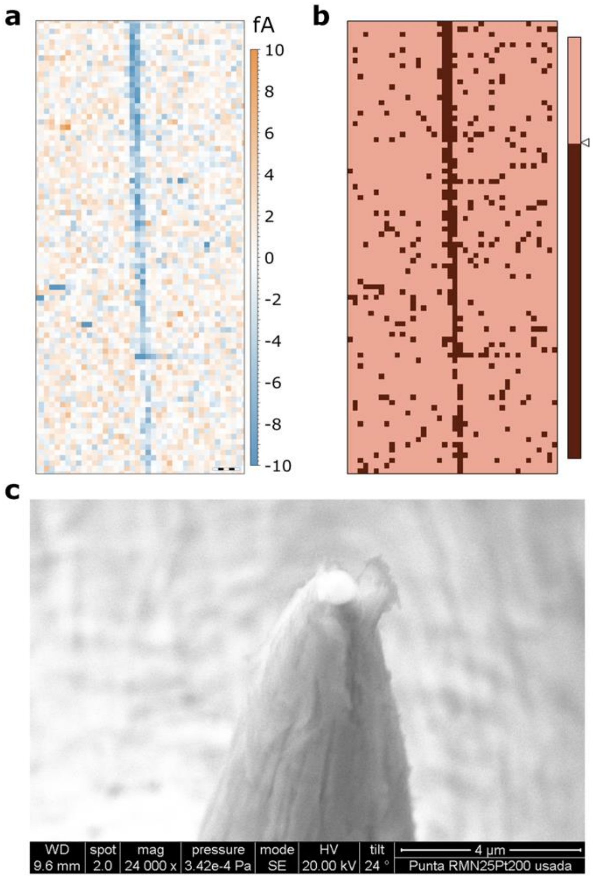

Supplementary Figure 4: Probe shape after scanning a DPFM-So image obtained by digitally zoomed Figure 3b. The effect of the tip size on the measurements has been studied by analyzing Figure 3 of the main manuscript. After the zoomed in, an image filter was applied to the image in order to obtain a binarized image of the data, scale bar 1 μm, **b**. With such image, we found, statistically, that the mean thickness of the line is 297 nm. **c** SEM image of the tip used along all the measurements of the present work. The increased thickness of the line is partially due to tip degradation, but also to the nanoindentation of Lithium Niobate by the tip. The tip material, platinum, and the sample, Lithium Niobate, have similar Young modulus, 168 GPa and 170 GPa, respectively. In order to reveal tip degradation after all the images, we performed SEM images of the tip in order to measure its tip radius, 144 nm, in good agreement with the average current line width of **b**.

Supplementary Note 1: Electrical power generated

The electrical power generated by the direct piezoelectric effect can be estimated through the use of Ohms law and the feedback resistor value, as denoted by the expression:

$$P = I^2 * R_f \quad (1)$$

Where R_f is the transimpedance amplifier feedback resistor of 10^{12} Ohms and I is the current recorded by the amplifier. Such approximation can be performed if the input impedance of the voltage amplifier stage is high enough, which in our case is 6,6 x 10^6 Ohms. Through this equation we can find the piezopower generated at DPFM-Si frame (**Supplementary Figure 5**) and DPFM-So. Both random profiles obtained from each of the images where clear differences are observed between the electrical power generated at domain walls and within domains .

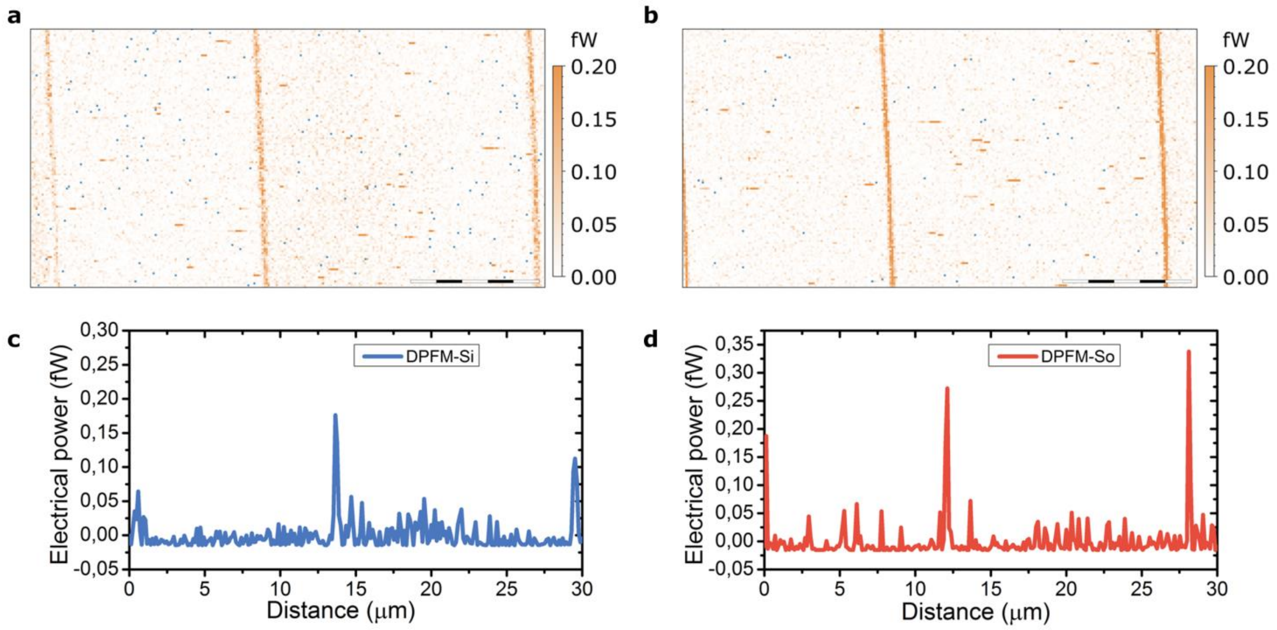

Supplementary Figure 5: Electrical power generated a. Piezopower generated calculated from DPFM-Si and **b** Piezopower generated calculated from DPFM-So images, scale bar 7,5 μm. **c** and **d** show random selected profiles froma and b, respectively, in order to see the differences between the piezopower generation at domain walls and at a single domain.

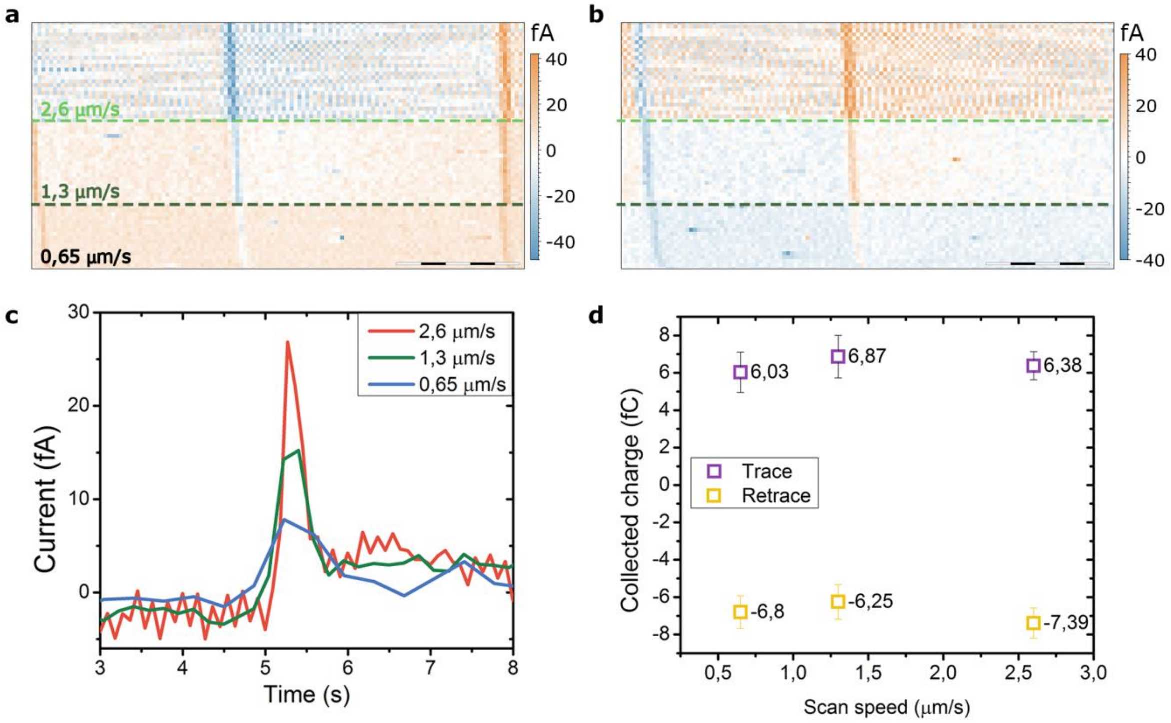

Supplementary Figure 6: Charge dependence of tip speeda, DPFM-Si image and **b**, DPFM-So image of the PPLN test sample acquired at different proposed speeds of 0.65, 1,3 and 2,6 μm s^{-1}, green lines denoted the area for each speed, scale bar 7,5 μm. Current vs Time graph, **c** extracted from the DPFM-Si image, center line, which corresponds to the current generated while scanning at the different speeds proposed. **d**, Collected charge (fC) vs Scan speed (μm s^{-1}) for each of the proposed velocities, which shows that there is no correlation between tip speed and charge generated, concluding that the charge scrapping process is not present at this range of speed.

Supplementary Note 2: Cantilever spring constant

In order to obtain an accurate value of the force applied to the sample we measured the specific dimensions of the cantilever used throughout the measurements in the manuscript. From the tip dimensions, we used the following formula, which was provided by the tip manufacturer:

$$k = \frac{EWt^3}{4L^3} (\mathrm{N\ m^{-1}}) \quad (2)$$

Where:

E is the young modulus of platinum (168,000,000,000 N m^{-2})
W is the measured cantilever width (m)
L is the length of the cantilever (m)
t is the tickness of the cantilever which is calculated as follows:

$$t = \frac{pir^2}{W} (\mathrm{m}) \quad (3)$$

Where r is the radius of the wire that is used to produce the cantilever, 0,0000125 m

The cantilever was analyzed through an optical microscope, side view and top view. Using the measured units and with the above formulas, we that the K constant of the cantilever was 184 N m^{-1}. The nominal spring value provided by the manufacturer was 250 ± 100 N m^{-1}. By performing the above calculations and measurements, the error for such value can be reduced to be ± 7 N m^{-1}. We have supposed a measurement error for optical microscopy images of ± 1 μm for the length error, e_L and width error, e_W . The thickness error is calculated as:

$$e_t = \frac{pi\, r^2}{W^2} e_W = 1{,}4\ 10^{-7}\ (\mathrm{m}) \quad (4)$$

By error propagation, we can use the following formula to calculate the error for the cantilever spring constant:

$$Error = \sqrt{\left(\frac{3e_L EWt^3}{4L^4}\right)^2 + \left(\frac{3e_t t^2 EW}{4L^3}\right)^2 + \left(\frac{e_W t^3 E}{4L^3}\right)^2} = \sqrt{7{,}8 + 21{,}6 + 9{,}8} = 6{,}2 \text{ N per m} \quad (5)$$

The final value for the cantilever spring constant is then found to be 184 ± 7 N m^{-1}.

The specific deflection sensitivity of the cantilever used is found by performing several force vs distance curves like the one showed in **Supplementary Figure 7**. From the linear part, deflection sensitivity constant is obtained from a linear fitting and so the specific deflection signal-which is in voltage units- can be converted to nm units. With such linear fit, we found that the calibration constant is 401 nm V^{-1} and 403 nm V^{-1} for approach and retract curves respectively. The error for such linear fittings are not considered since it is about ± 3 nm, which represents less than 1% error. The spring constant of the cantilever used remains the same for all the measurements, since it is exactly the same probe. However, the deflection vs distance curve has to be performed each time that the cantilever is placed in the holder. **Supplementary Figure 7** only shows an example of the curve used to calibrate the deflection sensitivity.

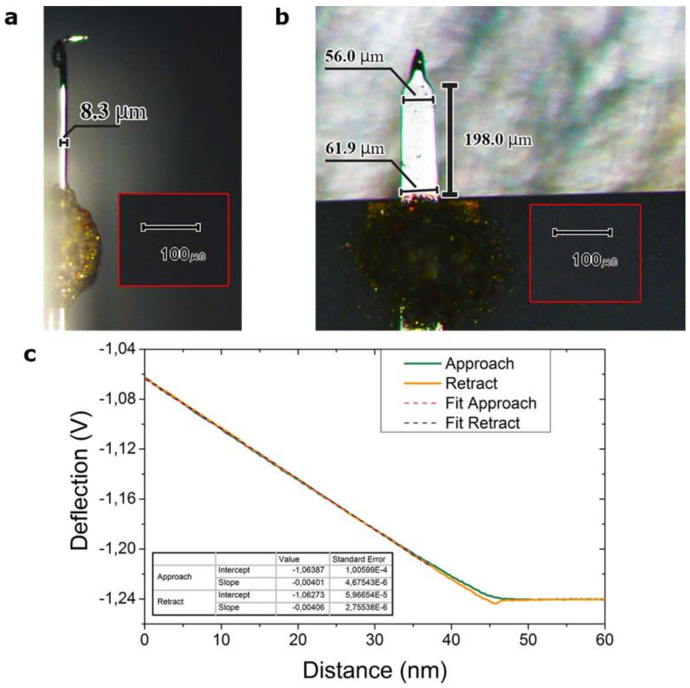

Supplementary Figure 7: Cantilever calibration. a and **b** optical microscopy images of the cantilever used, which corresponds to the side and top view, respectively. The images were used to obtain the specific size of the cantilever used along all the measurements. **c** Deflection vs Distance curve obtained through approaching and retracting the tip to the surface. A linear fitting is used to obtain the specific deflection sensitivity constant before starting measurements.

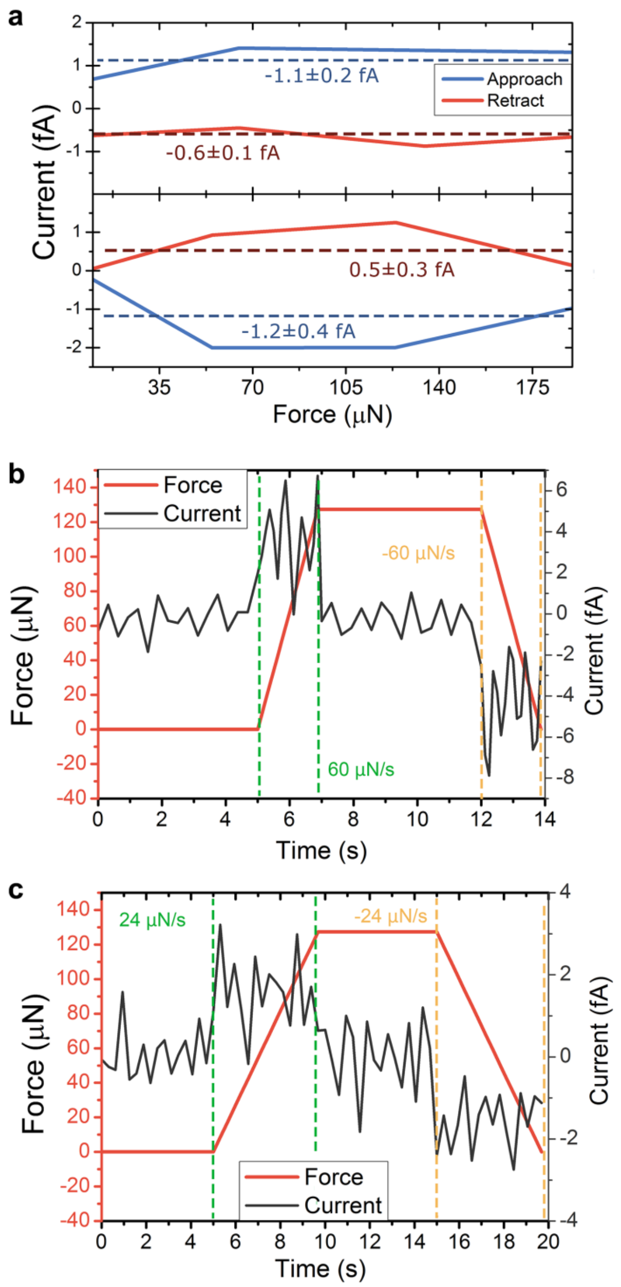

Supplementary Figure 8: Spectroscopy experiments. a, corresponds to the Figure 4f of main manuscript on a PPLN single crystal, where an average current was calculated and plotted as a dotted line. We can now use the sweep rate of the force applied, 53 μN s^{-1}, to estimate the d_{33} values by averaging the current read by the amplifier and dividing by the force sweep rate with results 20 ± 4, 11±3, 9±6 and 22±10 pC N^{-1}. The errors obtained correspond to the statistical errors and the additional error of the electrical calibration process and force calibration process that it was discussed in the manuscript. In spite of the fact that the average value, 15±6 pC N^{-1} is inside the error bars obtained for the imaging approach. **b** and **c**, corresponds to a Force (red) and Current (black) vs Time plot performed at two different force sweep rates, of 60 μN s^{-1} and 24 μN s^{-1} on the BFO film. It can be seen that the current depends on the specific force sweep rate applied. For the case of the BFO sample, the force sweep rate applied for b, is very similar, 60 μN s^{-1}, to the case of the Periodically Poled Lithium Niobate. However the current recorded by the amplifier is 3.7 ± 0.7 and -4.5 ± 0.8 fA. We can calculate the d_{33} values for such measurements by dividing such current between the force sweep rate, which gives 61±14 and 75±17 pC N^{-1}.

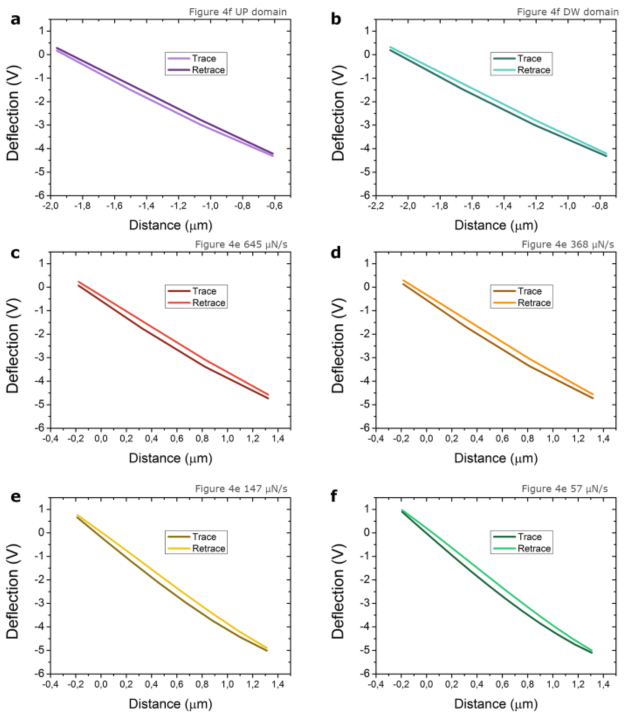

Supplementary Figure 9: Spectroscopy curves. Deflection (V) vs Distance (μm), **a** corresponds to the curve of Figure 4f UP domain, **b** corresponds to the curve of figure 4f DOWN domain, **c** corresponds to the curve of figure 4e at 645 μN s^{-1}, **d** corresponds to the curve of figure 4e at 368 μN s^{-1}, **e** corresponds to the curve of figure 4e at 147 μN s^{-1} and **f** corresponds to the curve of figure 4e at 57 μN s^{-1}. The spectroscopy experiments were carried out by recording normal Force-vs-Distance curves, while at the same time, we recorded the ADC channel to acquire the signal from the amplifier. In order to make clearer the reproducibility of such curves, we incorporated in this supplementary information all the Force-vs-Distance curves that correspond to the Curves of Figure 4e and 4f of main text. We did not include the current channel, as it is included in the figures at the main text. Within this data we can see the reproducibility of the curves along each of the different force sweep rate applied.

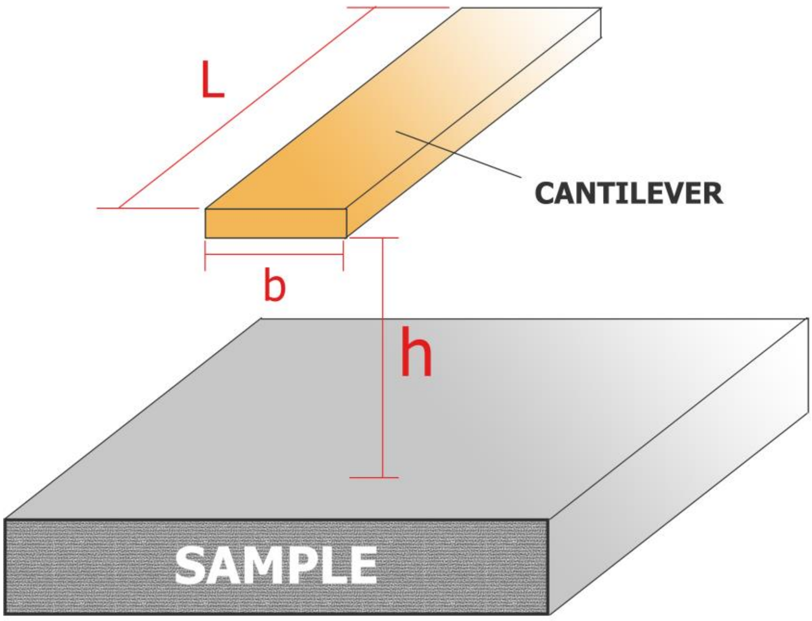

Supplementary Figure 10: Camtilever-sample capacitance model. Scheme of the Cantilever and sample setup used to calculate the tip-sample capacitance as a parallel plate capacitor.

Supplementary Note 3: Cantilever-sample capacitive coupling

In order to confirm that the current collected in spectroscopy measurements is related to the piezoelectricity of the sample and to exclude artifacts from sample-cantilever capacitive coupling, we performed a calculation of the displacement currents for the cantilever. With such calculation, we want to obtain the current due to the changes of the cantilever-sample capacitance during spectroscopy curves. We modeled the cantilever and sample as a parallel plate capacitor.

We used the following parameters, L = 200 µm, b= 35 µm, h= 80 µm and dielectric constant, ε = 8,8 10^{-12} F m^{-1}. If we apply the known capacitance expression for a parallel plate capacitor, we can find:

$$C = \varepsilon \, \frac{A}{h} \quad (6)$$

Where A is the area of the capacitor, in our case

$$A = L * b \ (7)$$

Performing the calculation, we obtain that the capacitance is 7,7 10^{-16} F.

The previous calculations correspond to the initial state of the lever. We now calculate the capacitance at the end of the spectroscopy curve, by assuming that the whole cantilever approaches to the sample surface by 5 µm, so the new parameters are L = 200 µm, b= 35 µm, h= 75 µm, ε = 8,8 10^{-12} F m^{-1}.

With such parameters, the capacitance is now 8,2 10^{-16} F.

The capacitance variation is now:

$$\Delta C = 5{,}13\ 10^{-17}\ F$$

The voltage difference between the capacitor plates stores a charge in the capacitor. We use here a value of 3 Volts, an approximation of the work function difference between the plates. In reality we are always in contact with the material, so such 3V value is an overestimation of the voltage difference. With such parameters, we can calculate the charge as:

$$\Delta Q = \Delta C * V = 1{,}54\ 10^{-16}\ C.$$

Such charge, if measured in 3 seconds (the time employed for curves of Figure 4f), will produce a current of 0,05 fA which is almost two orders of magnitude smaller than the experimentally recorded currents.

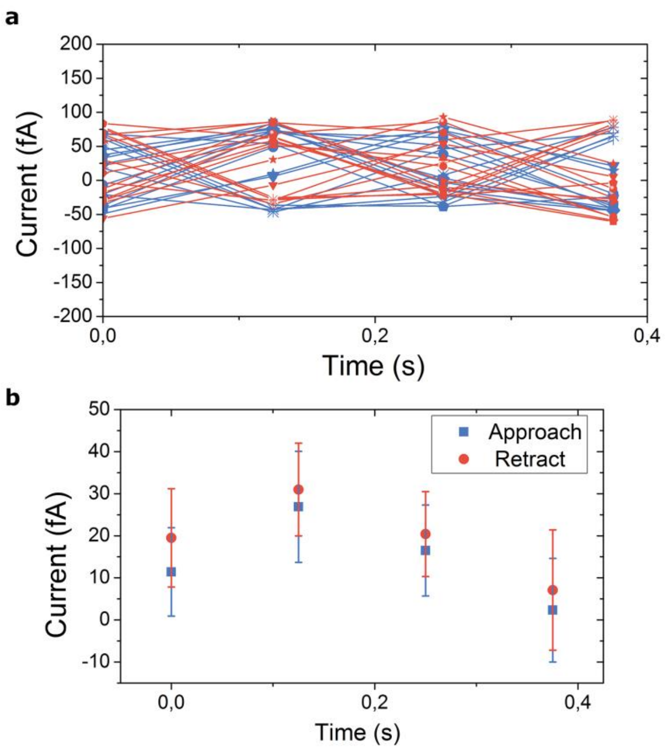

Supplementary Figure 11: Spectroscopy experiments in glass.a, Current (fA) vs Time (s) obtained for a glass slide test sample and **b** Current (fA) vs Time (s) where we average all the curves. There is no difference between the approach and the retract curves for such series of measurements confirming that the displacement current is not measurable by the amplifier. In order to increase the current induced by capacitive coupling, we used the extreme case where the Z piezo range is 10 µm. This experiment was carried out with the transimpedance amplifier populated with a 10GOhm resistor which boosts the bandwidth of the overall system to 159 Hz. Such change also increases the current noise of the amplifier in one order of magnitude as well as increasing the 50Hz noise pickup. From our data, it is not possible to distinguish which are the approach or the retract curves, confirming that the displacement current due to the changes in the sample-cantilever capacitive coupling cannot be measured by the amplifier.

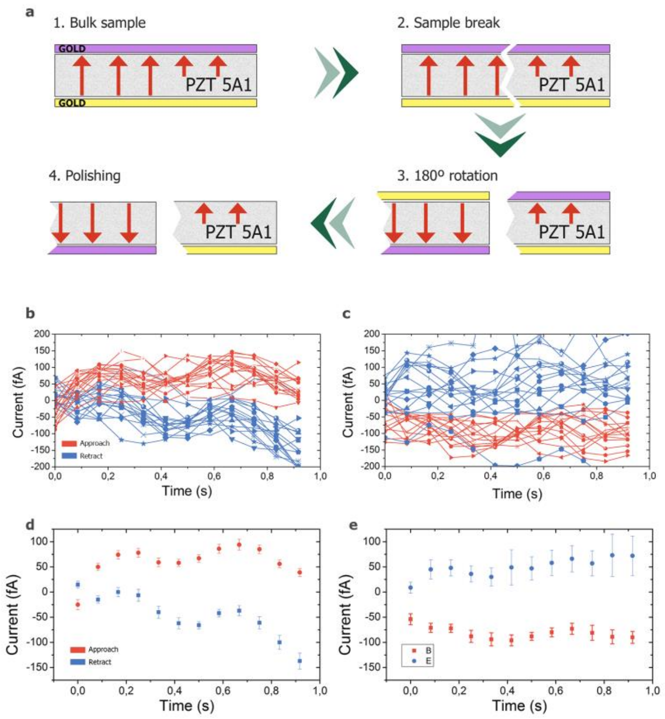

Supplementary Figure 12: Spectroscopy experiments in PZT a scheme showing the preparation of the test samples. The poled piezoelectric sample was broken in half, one of the halves was flipped 180º and both top electrodes were removed by polishing in order to uncover the ceramic layer underneath. **b** Current (fA) vs Time (s) curves performed in a full Approach and Retract cycle, where the approach cycle is depicted in red color, while the blue one corresponds to the retract cycle. Figure **c** Current (fA) vs Time (s) which corresponds to the down polarized sample. Note that, as expected from the direct piezoelectric effect, the current profiles of the approach and retract curves corresponding to opposite dielectric polarizations present inverted signs. **d** Current (fA) vs Time (s) for the average over multiple curves obtained for **b** and **e** Current (fA) vs Time (s) by averaging curves of **c**.

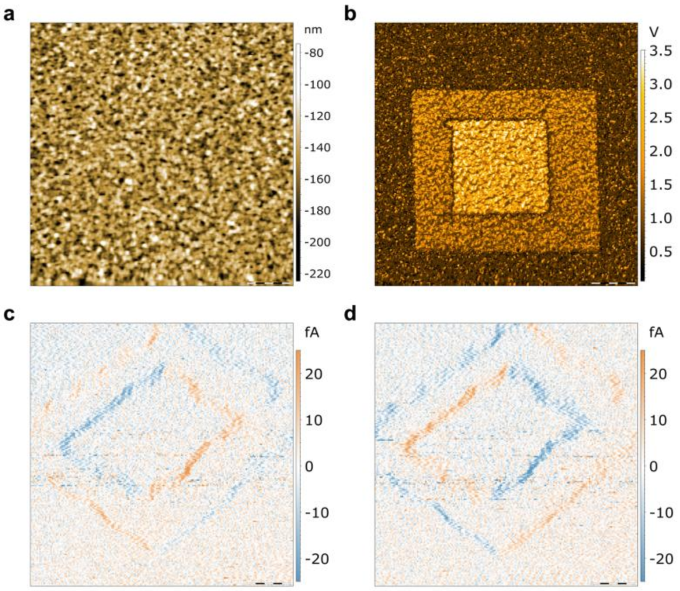

Supplementary Figure 13: Rotation of imaging angle. a, topography and **b** PFM amplitude image obtained in PFM mode by using a RMN-25Pt300 tip, with an applied load of 4 μN, scale bar 2,5 μm. **c**, DPFM-So and **d**, DPFM-Si of the BFO test sample, where the recorded pattern was read with a scan direction angle of 45º, scale bar 2,5 μm. The images show that the current motifs are also rotated, discarding electronic signal crosstalk artifacts.

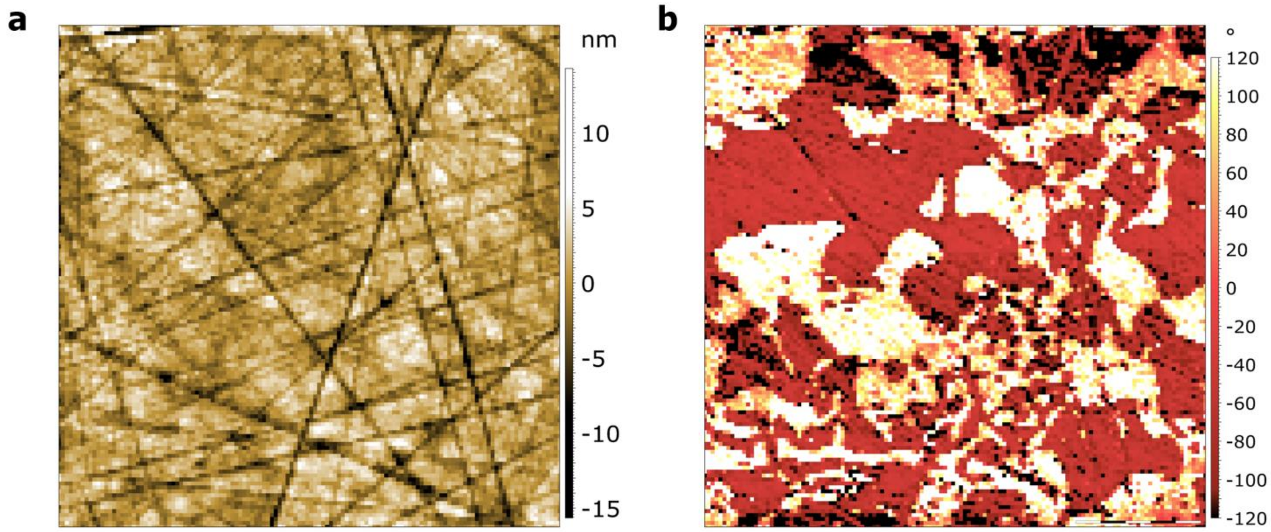

Supplementary Figure 14: Natural domains of PZT. a, AFM topography and **b**, PFM phase image of the proposed PZT sample, scale bar 2,5 µm. Effects of the poling process can be seen on the topography images, while the natural domains can be distinguished into the PFM phase signal.

Supplementary Note 4: Comparison with Charge Gradient Microscopy

Due to the apparent similarities between DPFM images and CGM images, we discuss below different aspects and present some calculations corroborating that DPFM can map the piezo electric charge generation at the nanoscale:

1. Collected charge versus applied pressure.

   In the CGM manuscript a graph between the deltaCurrent, defined as the top positive peak current minus the bottom peak negative current, is plotted as a function of pressure. We represented their data and our measurements in a graph, see Supplementary Figure 15.

By plotting both sets of CGM data and DPFM data, we can immediately see that the relation between the applied pressure and the current collected are related to different physical quantities. It can be seen that the current recorded in CGM is almost 300 times higher than in the case of DPFM mode. The pressure applied to the material is one order of magnitude higher in DPFM than in CGM, assuming a contact area of radius of 148.5 nm.

More importantly than the numbers itself, is the behavior of the curves. According to Hong et al. [2], the surface charge scrapping process is saturated at a certain pressure which was calculated to be ~300 MPa. Above this pressure, collection of scraped does no longer occur and hence, the current will not increase. However, for our data, we applied pressures up to 3376 MPa and obtainedincreasing currents from the first pressure point. The DPFM data, black line of Figure S15, cannot be explained by the CGM model, in which the current does not increase above 300 MPa.

2. No charge collected with 9 μN (130 MPa)

   In the the work of Hong et al, said it is stated that for a Periodically Poled Lithium Niobate, an applied force of 1 μN, which corresponds to a pressure of 120 MPa, is enough to fully remove the screening charge of the material[1]. This is used determine a collected charge of 10fC. Following the same arguments, if the measurements presented in this work were CGM one should expect some current being produced in the 9 μN region of Figure 3a and 3b of main manuscript, in contrast to our measurements. In such region, the pressure exerted, assuming a contact area of 148,5 nm of radius, is 130 MPa. If we are reading a charge scrapping mechanism equivalent to that reported by Hong et al., we should collect a charge of 10 fC.

3. Estimation of surface charge collected by our tip.

   Following the calculations presented by Hong et al., we estimated the surface charge that our tip can collect. From **S4** we can estimate the area which is in contact with the material under study, as:

   $A = \pi \cdot r^2 = 69277\ \text{nm}^2$

   So the surface screening charge that we should collect is:

   $C = A * \rho = 6.93 \cdot 10^{-10}\ \text{cm}^2 \cdot 8 \cdot 10^{10}\ \text{fC cm}^{-2} = 55.4\ \text{fC}$

where ρ is 80 μC cm^{-2} and corresponds to the surface screening charge of Lithium niobate.

However, the maximum charge we could collect was 5 fC, at a pressure of 3376 MPa. Such results are not consistent with a processes of scraping of surface screening charges. If we are collecting surface charge rather than the generated piezoelectric charges we should measure 55fC instead of 5 fC.

4. PPLN charge is 5fC, BFO charge is 25 fC and PZT charge collected is 90fC.

We specifically selected a BFO material, because it has the same surface screening charge than PPLN. From the data presented in the manuscript, we collected a total charge of 25 fC from the BFO material, as the charge read by PPLN was 5fC. If both materials have the same surface charge density, it is obvious that the larger charge collected for BFO cannot come from a different surface charge mechanism. More remarkable is the case of PZT, in which the surface screening charge is significantly lower than ine PPLN and BFO. Such decrease in surface charge should give a much lower value of the collected charge. However the charge collected is for PZT is one order of magnitude higher than for Lithium Niobate. Interestingly, the collected charges for the different tested materials are in good correlation with their corresponding piezoelectric coefficients, in agreement with the predictions of a process of piezogenerated charges.

5. Spectroscopy on BFO

In order to elucidate if the current signal really depends on the applied force sweep rate, we performed several experiments with different force profiles, see figures S8b and S8c. We started from a given force, which is considered as the 0 force value, and we recorded the currents obtained while applying several force profiles to the material. It can be seen, Figure S8b, that when the force increases at a rate of 60 $\mu N\ s^{-1}$ we get an average current of nearly 4fA. If the rate is reduced to 28 $\mu N\ s^{-1}$ the current recorded scales accordingly. A charge scraping mechanism cannot, by any means, account for the observed dependence of the current on the force sweep rate.

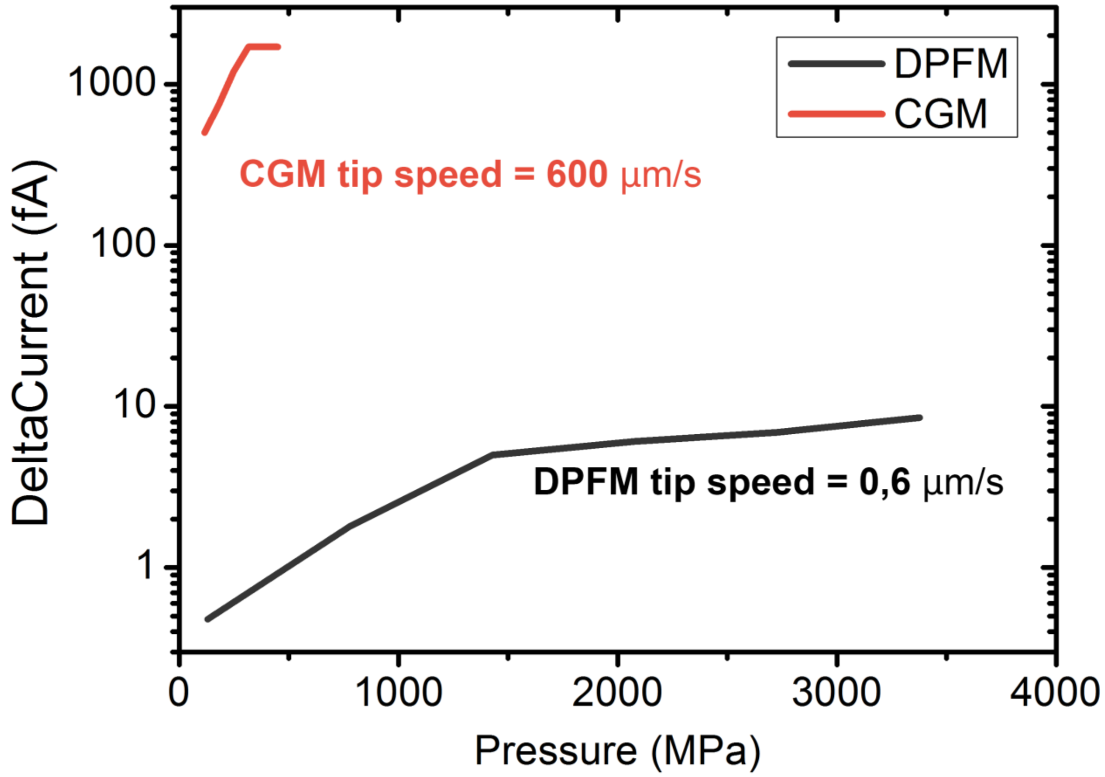

Supplementary Figure 15: CGM vs DPFM comparison, DeltaCurrent (fA) vs applied Pressure (MPa) comparison between data extracted from Hong et al PNAS article versus our experimental data. The pressure applied was obtained with the use of our measured tip radius which is 148,5 nm. Y axis is plotted in logarithmic scale for ease clarification.
